# Anxiety amongst physicians during COVID-19: cross-sectional study in Pakistan

**DOI:** 10.1186/s12889-020-10134-4

**Published:** 2021-01-11

**Authors:** Qaisar Khalid Mahmood, Sara Rizvi Jafree, Aisha Jalil, Syed Mujtaba Hasnain Nadir, Florian Fischer

**Affiliations:** 1 Department of Sociology, International Islamic University Islamabad, Islamabad, Pakistan; 2 Department of Sociology, Forman Christian College (A Chartered University), Lahore, Pakistan; 3 School of Integrated Social Sciences, University of Lahore, Lahore, Pakistan; 4 Health Education England, North West Deanery, UK; 5 Institute of Public Health, Charité – Universitätsmedizin Berlin, Berlin, Germany; 6 Institute of Gerontological Health Services and Nursing Research, Ravensburg-Weingarten University of Applied Sciences, Weingarten, Germany

**Keywords:** Coronavirus, Pandemic, Medical doctor, Physician, Healthcare, Practitioner

## Abstract

**Background:**

Ensuring safety and wellbeing of healthcare providers is crucial, particularly during times of a pandemic. In this study, we aim to identify the determinants of anxiety in physicians on duty in coronavirus wards or quarantine centers.

**Methods:**

We conducted a cross-sectional quantitative survey with an additional qualitative item. Five constructs of workload, exhaustion, family strain, feeling of protection, and anxiety were measured using items from two validated tools. Modifications were made for regional relevance. Factor analysis was performed showing satisfactory Cronbach alpha results. Overall, 103 physicians completed the questionnaire.

**Results:**

T-test results revealed significant associations between gender and anxiety. Structural equation modeling identified that high workload contributed to greater exhaustion (β = 0.41, R^2^ = 0.17, *p* < 0.001) and greater family strain (β = 0.47, R^2^ = 0.22, *p* < 0.001). Exhaustion (β = 0.17, *p* < 0.005), family strain (β = 0.34, *p* < 0.001), and feelings of protection (β = − 0.30, *p* < 0.001) significantly explained anxiety (R^2^ = 0.28). Qualitative findings further identified specific needs of physicians with regard to protective equipment, compensation, quarantine management, resource allocation, security and public support, governance improvement, and health sector development.

**Conclusions:**

It is imperative to improve governmental and social support for physicians and other healthcare providers during the corona pandemic. Immediate attention is needed to reduce anxiety, workload, and family strain in frontline practitioners treating coronavirus patients, and to improve their (perceptions of) protection. This is a precondition for patient safety.

**Supplementary Information:**

The online version contains supplementary material available at 10.1186/s12889-020-10134-4.

## Background

The success in controlling the spread of the coronavirus differs between countries, based upon their strategies for infectious disease management and the strength of healthcare systems [1]. Pakistan has a comparatively weak public health system and has shown a slower response mechanism to deal with the unprecedented emergency of the COVID-19 pandemic [2]. The global response for combatting COVID-19 was mainly based on approached related to physical distancing and lockdown [3]. However, the effectiveness of these actions and prevention of spread is not consistent or certain, causing great anxiety and fear among physicians. As frontline practitioners and custodians for patient treatment and recovery, they are suffering innumerable and unknown challenges. It is also known that when physicians are not confident and secure they are less able to deliver health care at the highest level [4, 5].

The outbreak of the coronavirus pandemic in Pakistan was in February 2020 and the first nation-wide lockdown was initiated in March 2020. Total cases have been recorded at nearly 0.4 million and currently, as at November 2020, Pakistan has entered its second wave of coronavirus peak with semi-lockdown in hot spots across the nation. The country, with a population of 212 million, is known for its resource shortages and low doctor to patient ratios at 1:1300 [6].

Many healthcare practitioners have remained on duty in coronavirus wards, isolations centers and emergency departments, serving both infected and non-infected emergency patients. Several known challenges towards health and wellbeing are faced by physicians locally since the outbreak of the coronavirus, such as stress of performing with scarce resources [7], burden of long working hours [8], inadequate support and training from administration and employers [7], stigma and exclusion from colleagues [9], anxiety due to shortage of protective gear provision [10], and fear due to abuse and violence from the public [11].

Globally, healthcare organizations and governments are striving to protect physicians from COVID-19. Protocols for sterilization, safety, cleaning, and disinfecting in hospitals, isolation centers, and other health center spaces are being communicated and enforced [12, 13]. This is of critical importance, as infected physicians, and other healthcare providers, would mean a decrease in trained workforce for dealing with the pandemic, which no country can afford [14]. Furthermore, it is important to monitor the psychological needs of physicians in times of pandemics, specifically in relation to their anxiety level as it related to patient safety [15, 16]. It is expected that having to adapt with a new work environment in quarantined wards may lead to stress among healthcare personnel [17].

Previous research suggests that anxiety levels of physicians are influenced by high workload and role strain [18]. During pandemics excessive workload can also lead to high rates of exhaustion and intentional absence from work [19]. When physicians are dissatisfied with workplace safety and have greater fears of exposure to infection there is also less commitment for patients [16]. Additionally, an increase in workload prevents time and energy for family, especially young children and elderly dependents like aging parents [20]. Higher anxiety in physicians is also associated with fears of exposing families and children to infections after returning from work [21]. A recent study shows that anxiety in physicians during the coronavirus outbreak is related to perceptions of inadequate and insufficient protection by the employer and state [22].

The mental health of physicians on duty for coronavirus infected patient’s needs immediate investigation. Given that there is no empirical evidence in this area yet, we aimed at identifying determinants of anxiety in physicians serving COVID-19 patients in Pakistan. The following hypotheses are tested within this study:

- H1: Higher workload in physicians in times of COVID-19 creates (i) greater exhaustion, (ii) greater family strain, and (iii) reduction in feelings of protection.
- H2: Levels of anxiety in physicians in times of COVID-19 are positively influenced by (i) greater exhaustion, (ii) greater family strain, and (iii) reduced feelings of protection.

## Methods

### Study design

We conducted a cross-sectional online-based survey in order to secure social distancing during the coronavirus pandemic. Written informed consent was taken from all participants. No names were asked from participants and data related to hospital affiliation has not been reported to secure anonymity and confidentiality.

### Sample

The selection criterion for this study was medical doctors of Pakistan serving in (i) coronavirus wards or (ii) coronavirus isolation centers. We avoided errors in sampling and sampling bias through the following measures: (i) requesting hospital administrations to communicate physicians working in coronavirus wards through email or link sent through text message, (ii) directly contacting doctors we knew were working in coronavirus wards or isolation centers through shared author network via WhatsApp, and (iii) messaging physician groups on Facebook requesting for response from physicians working in coronavirus wards or isolation centers and then sending the survey to people who responded. Furthermore, we made information about sociodemographic characteristics and information about coronavirus ward or isolation center belonging and city mandatory before the survey questions started; thus, reducing potential problems of sampling error and bias [23].

### Study constructs

We developed a questionnaire for this study (Supplementary file 1) including questions related to sociodemographic characteristics of respondents and measured the following five main study constructs: workload, exhaustion, family strain, feeling of protection, and anxiety. The assessment of constructs was based on validated tools, which were taken from a study on the psychological impact of the H1N1 pandemic [24], and the Impact of Event Scale [25]. We modified questions for regional and situational relevancy. All items are based on statements and the respondents answered on a five-point Likert scale (“Never”, “Rarely”, “Sometimes”, “Often”, or “Always”). The items within a scale were summed without any weighting, with the lowest agreement (“Never”) being “1” and the highest agreement (“Always”) being “5”.

The following four items were used to measure workload: “I feel I have lack of knowledge about coronavirus infection”, “I feel I have incomplete knowledge about prevention from this virus”, “I feel I have no choice but to work due to obligation”, and “I feel hesitation in working”.

Exhaustion was measured using the following five items: “I am exhausted physically”, “I feel burdened by the changed nature of work”, “I am exhausted mentally”, “I feel burdened by the increase in quantity of work”, and “I have insomnia”.

We measured family strain with the following three items: “I am worried I carry the virus without symptoms, and place my family (children and parents) at risk”, “I am worried about returning home and exposing my children to the virus”, and “I cannot stop worrying for my family whenever I see/treat a patient in critical situation”.

The feeling of protection was measured using the following items: “I feel I am protected by my hospital administration”, “I feel I am protected by the federal government”, and “I feel I am protected by security forces”.

Anxiety was measured by the following items: “I feel anxious about being infected during commuting/travel to work”, “I feel anxious about compensation, in the case of being infected”, and “I feel anxious about being infected by the virus”. Reliability analysis revealed satisfactory to high Cronbach’s Alpha results for each of the five scales (Table 1).

**Table 1:** Reliability analysis and scales descriptives

Variables	α	Mean	SD	Min.	Max.	N of items
Workload	0.734	10.83	3.85	4	20	4
Exhaustion	0.874	14.10	5.40	5	25	5
Family strain	0.817	12.92	2.84	4	15	3
Feeling of protection	0.799	7.49	3.23	3	15	3
Anxiety	0.663	10.76	2.98	3	15	3

Furthermore, the survey included three open-ended questions to allow medical doctors to list their needs from their employers, government, and community.

Cronbach’s Alpha results for all study constructs were satisfactory: (i) workload (α = 0.725), (ii) exhaustion (α = 0.878), (iii) family strain (α = 0.791), (iv) feeling of protection (α = 0.796), and (v) anxiety (α = 0.644) (Supplementary file 2).

### Data collection

Data was collected from April 1 to May 6, 2020. We messaged five Facebook groups for doctors working in Pakistan, contacted 35 hospitals listed as having coronavirus wards via e-mail [26], and messaged multiple Whatsapp groups through author’s networks. We followed up our messages and e-mails with two follow-up reminders at six-day intervals, based on empirical evidence regarding average response time for online surveys of 5.59 days [27]. We were finally able to recruit 103 physicians who completed the survey. Though we reached out to multiple social media groups and e-mail networks of physicians, we received a low response. We believe this is due to great work pressure and less time for survey response of physicians during critical times. Based on a review of findings and especially the qualitative data, we identified critical factors for policy mobilization and thus chose to stop data collection despite low response in order to disseminate results. There is no agreed-upon standard for a minimum acceptable response rate in social research and no agreement about recommended calculation for online survey response [28]. Though some researchers may choose to calculate a response rate based on how many people viewed their survey and partial submission, we assigned settings to our survey to only accept complete submission and did not collect data for viewership.

### Data analysis

We used SPSS and its module AMOS for data analysis. Descriptive statistics were used to analyze the sample. Independent sample t-tests were performed to assess the relationship between study variables and sociodemographic characteristics. Pearson correlation was used to investigate the association between study variables. Finally, a structural equation model was applied for examining the relationship between the dependent variable (anxiety) and independent variables (workload, exhaustion, family strain, and feeling of protection). The significance level was assigned at 95% for all tests.

For the qualitative responses we conducted a thematic content analysis. We grouped all statements in categories dealing with the needs emphasized by the physicians. Overall, we were able to identify seven areas with this inductive approach.

## Results

### Study variables and sociodemographic characteristics

Our sample (*n* = 103) consists of 51.5% (*n* = 53) female physicians. The majority is above 30 years of age (*n* = 71, 68.9%) and has been working in the clinical setting for up to 5 years (*n* = 73, 70.9%) (Table 2).

**Table 2:** Sociodemographic characteristics of respondents (*n* = 103)

Variables	n	%
Gender		
Female	53	51.5
Male	50	48.5
Age		
Up to 30 years	71	68.9
31 years and above	32	31.1
Work experience		
Up to 5 years	73	70.9
6 years and above	30	29.1

Independent sample t-test between sociodemographic characteristics and study constructs have been applied (Tables 3, 4, 5). Results reveal a significant association only between gender and anxiety. Women (M = 13.66, SD = 2.157) face significantly higher anxiety levels compared to men (M = 12.26, SD = 3.036) (t (101) = − 2.71, *p* = 0.008).

**Table 3:** Independent sample t-test between age and study variables

Variables	Age	Mean	SD	t	***p***-value	95% CI	
						Lower	Upper
Workload	Up to 30 years	10.94	3.573	0.277	0.783	−1.388	1.838
	31 years and above	10.72	4.320				
Exhaustion	Up to 30 years	14.54	5.280	1.181	0.240	−0.917	3.612
	31 years and above	13.19	5.538				
Family strain	Up to 30 years	10.79	2.893	0.061	0.951	−1.215	1.292
	31 years and above	10.75	3.132				
Feeling of protection	Up to 30 years	7.24	3.007	−1.306	0.195	−2.231	0.460
	31 years and above	8.13	3.554				
Anxiety	Up to 30 years	12.75	2.907	−1.315	0.191	− 1.890	0.383
	31 years and above	13.50	2.125

**Table 4:** Independent sample t-test between gender and study variables

Variables	Gender	Mean	SD	t	***p***-value	95% CI	
						Lower	Upper
Workload	Male	10.88	3.287	0.016	0.987	−1.482	1.506
	Female	10.87	4.261				
Exhaustion	Male	13.46	5.195	−1.208	0.230	−3.372	0.820
	Female	14.74	5.509				
Family strain	Male	10.46	2.772	−1.058	0.293	−1.770	0.539
	Female	11.08	3.112				
Feeling of protection	Male	7.90	3.315	1.191	0.236	−0.498	1.996
	Female	7.15	3.066				
Anxiety	Male	12.26	3.036	−2.711	0.008	−2.425	−0.376
	Female	13.66	2.157

**Table 5:** Independent sample t-test between work experience and study variables

Variables	Experience	Mean	SD	t	***p***-value	95% CI	
						Lower	Upper
Workload	Up to 5 years	10.84	3.671	−0.158	0.875	−1.774	1.512
	6 years and above	10.97	4.165				
Exhaustion	Up to 5 years	14.59	5.359	1.399	0.165	−0.678	3.922
	6 years and above	12.97	5.314				
Family strain	Up to 5 years	10.97	2.779	1.051	0.296	−0.597	1.943
	6 years and above	10.30	3.344				
Feeling of protection	Up to 5 years	7.41	3.063	−0.511	0.610	−1.736	1.024
	6 years and above	7.77	3.540				
Anxiety	Up to 5 years	13.00	2.593	0.113	0.910	−1.101	1.234
	6 years and above	12.93	2.993				

### Correlation analysis

The findings in Table 6 indicate that workload is not correlated with anxiety. On the other hand, workload is positively correlated with exhaustion (r = 0.412, *p* < 0.01) and family strain (r = 0.473, *p* < 0.01). Similarly, anxiety is positively correlated with exhaustion (r = 0.370, *p* < 0.01) and family strain (r = 0.417, *p* < 0.01), and negatively correlated with feelings of protection (r = − 0.337, *p* < 0.01).

**Table 6:** Correlation analysis

Variables	Workload	Anxiety
Workload	1	0.179
Exhaustion	0.412^*^	0.370^*^
Family strain	0.473^*^	0.417^*^
Feeling of protection	−0.143	−0.337^*^
Anxiety	0.179	1

* *p* < 0.01

### Structural equation model

**Fig. 1:**
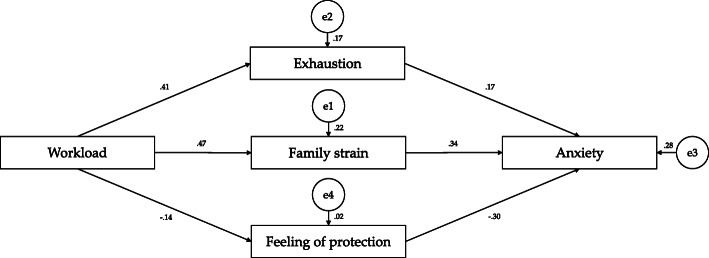
Structural equation model showing standardized estimates of path analysis

Path analysis reveals significant results for almost all observed study variables, except for the path between and workload and feelings of protection. In terms of the first hypothesis, high workload contributed to greater exhaustion (β = 0.41, R^2^ = 0.17, *p* < 0.001) and greater family strain (β = 0.47, R^2^ = 0.22, *p* < 0.001). However, workload did not explain feelings of being protected. Regarding the second hypothesis, exhaustion (β = 0.17, *p* < 0.005), family strain (β = 0.34, *p* < 0.001), and feelings of protection (β = − 0.30, *p* < 0.001) significantly explained anxiety (R^2^ = 0.28) among the physicians (Fig. 1).

### Qualitative responses

We were able to identify seven areas of needs shared by physicians within the qualitative responses. Table 7 provides further statements related to specific problems within these areas of need.

1. Problems with personal protective equipment (PPE): Need for improvement in quality or completeness of PPE kits and training for safe usage.
2. Compensation and contracts: Need for improvement in financial compensation, health insurance, and permanent job security, with cessation of salary cuts and tax cuts.
3. Management at quarantine centers or wards: Need for improvement in quarantine management in terms of sterilization and screening, and limiting non-emergency and non-corona outpatients and family attendants.
4. Resource allocation: Need for improvement in resource allocation in terms of staffing, government funding, separation of hospitals for coronavirus patients, childcare support for working mothers, training for newly engaged house officers, and hostel accommodation for corona staff.
5. Security and public support: Need for better security and public support, with regard to threats from patients and family attendants, strict monitoring for entry of screened patients, and barring of political demands.
6. Governance improvement: Need for improvements in governance for public health maintenance, with physicians and other healthcare providers on planning committees for coronavirus management at federal and provincial level, to improve reporting, testing, and detection. Maintaining lockdown, improving public awareness and media communication, regular sterilization of public spaces, and sponsoring the disadvantaged like the poor and illiterate for health literacy and food security.
7. Health sector development: Need for development of physicians, healthcare providers and health sector overall, through training for team-building and emotional stability, increase in research for coronavirus, introduction of digital health services for patients to access at home.

**Table 7:** Areas of need indicated by physicians serving coronavirus patients

Area of need	Specific problems within area
Problems with personal protective equipment (PPE)	• Complete kits of sterile gloves, N95 mask, hazmat goggles, full face shields or visors, and long sleeve disposable fluid repellent gowns are needed • Complete PPE kit is needed in other wards and not just in corona wards and emergency wards • Monitoring of these kits is needed: Some kits have non-functional items and are not sterilized properly • Guidance and training on kit management is needed (for donning and doffing of PPE)
Compensation and contracts	• Stop salary cuts of doctors with penalization of private employers for salary cuts and contract violations • Introduce financial compensation for risky jobs • Introduce financial security for children and family if we die • Health insurance for all physicians and other healthcare providers • Introduce payment for overtime • Transfer contractual employees serving coronavirus patients to permanent contracts • Issue a tax relief on salaries for frontline physicians and other healthcare providers
Management at quarantine centers or wards	• Stop outpatients unless emergency cases • Install vents in hospitals and clinics • Install sanitizer and disinfectants at short intervals across hospital • Install shower tunnels at entrance and regularly sterilize hospital corridors, walls, premises • Increase testing of front-line medical professionals • Active screening of all people entering hospital • Introduce system for maintaining physical distancing at hospitals and clinics • Limit attendants with patients to only one • Improve shower & sanitization facilities for workers in the hospital for when they leave for home • Improve sanitary measures in hospitals, some wards have no running water for handwashing • Walk-through gate for patients
Resource allocation	• More workforce, specifically hospital staff, doctors, nurses, and paramedic staff • Child support for day care while I am at work • Have separate hospitals for coronavirus patients • Government should start paying for inpatients in terms of medications, food and everything • Proper training for newly engaged house officers before placing them on clinical duty • Provide sterilized hostel accommodation to all corona duty staff
Security and public support	• Increase security for doctors receiving threats from patients and family attendants • Increase security at entrance to ensure only screened patients reach us and only one family attendant is with each patient • Improve public support and awareness that we are humans trying to do the best job we can • Remove pressure of political maneuvers and demands for VIP servicing
Governance improvement	• More effective plan of action for containment with doctors on planning committee • Ban religious gatherings and congregations • Remove beggars from streets and place them in relief homes with corona monitoring • Strictly maintain lockdown • Improve the reporting and detection system and introduce a central record • Introduce strict testing criteria • Reduce prices of food and basic necessities; solve the food shortage problems by improving ration distribution • Control and regulate the panic created by social media and television • Regularly sterilize roads and public places • Improve public awareness campaigns, especially for poor and illiterate, with clear instructions, regarding coronavirus and infection management • Increase intake of immunity boosters in the diet
Health sector development	• Awareness sessions are needed for positive thinking and emotional stability • Reduce stigma and bullying against colleagues trying to maintain physical distance at workplace • Improve team-building and communication between leadership and junior physicians/ healthcare providers • Invest and fund research in production of vaccines and medications • Start digital healthcare services

## Discussion

The aim of this study was to understand the challenges and experiences faced by physicians working during the coronavirus pandemic. The first hypothesis of the study is partially supported in that higher workload is associated with greater exhaustion and greater family strain. However, it does not have a relationship with feelings of protection. With regard to our second hypothesis, we were able to prove that greater exhaustion, greater family strain, and reduced feelings of protection impact on levels of anxiety among physicians. Other research from the developed world [29], developing countries [30], and Pakistan itself [31] corroborate that physicians are suffering from anxiety during the pandemic.

Higher workload is a crisis these days across the world due to staff shortages, leading to long and stressful duties for critical patients [32]. Research has also identified that anxiety is caused by unsustainable workload and increasing uncertain nature of job [33–35]. Recruitment of non-practicing doctors to relieve the workplace resource burden is urgently needed. In times of the COVID-19 pandemic, there is also the option of employing last year trainee doctors in the clinical setting under the supervision of licensed physicians. Furthermore, due to cultural reasons, many women in Pakistan are medical graduates who are not working and could, therefore, be engaged in clinical settings as well [36]. Historically, Pakistan is known for under-hiring of medical doctors [37], which means there is a window for brining physicians into the workforce through incentivized hiring.

Unprecedented work demands and long duty hours may be contributing to family strain, but a predominant concern for physicians is safety and exposure of family members [38, 39]. Researchers from South Asia have demonstrated that the fear of catching the coronavirus or passing it to family members has an impact on health care personal’s ability to work under pressure and in emergency situations [40]. Introducing hostel accommodation for physicians, and other healthcare providers, serving coronavirus patients have to be offered. There is also a need for sterilization services for physicians, and other healthcare providers, before returning home, and regular testing services. Being able to protect the family can reduce anxiety among physicians [41]. We also recommend the provision of family insurance in case of death, which would provide physicians, and other healthcare providers, with increased security for their dependents and children. Family strain and physician anxiety may also be exacerbated due to the fears and mental health deterioration of family members [42].

Research suggests that exhaustion is not only caused by physical workload, but emotional labor [43]. Physicians have to face great physical and mental exhaustion during normal work conditions at the clinical setting [44]. However, during pandemics and unstable work conditions, exhaustion levels are compounded due to fear of an uncertain future. Likewise, unfair or inadequate allocation of resources and staffing causes anxiety and work imbalance [45]. It is also true that the stress of having to follow hand cleaning protocols and continuously fearing for personal safety are contributing to physical and mental exhaustion [46]. Unfortunately, the overall professional commitment is affected when physicians feel over-burdened working in high-risk conditions [47]. In lieu of this, we urgently recommend the increase in staffing and resources, with shorter working hours for physicians managing coronavirus patients.

Our results also suggest that female physicians are suffering from anxiety related to COVID-19 than their male counterparts. There may be several explanations for this. The first explanation refers to gender stereotypes, because women as nurturers and innate care providers may face more anxiety and stress for their patients and the uncertainty of their recovery [48, 49]. Secondly, in a patriarchal society such as Pakistan, women as mothers, daughters, and wives, have to resume care duties for the household, children, and family members when they return home and cannot self-isolate after returning from work, placing family at risk of infection [50]. This would also contribute to increased exhaustion for female physicians. Additionally, lack of symmetrical assistance in home management, due to traditional norms, may be leading to more family strain. Lastly, female doctors may face more workplace burdens and less protection and support due to gender imbalance, with there being more male doctors in absolute numbers and also more in supervisory or senior positions [51, 52]. We recommend more support from government and employers for female physicians during this pandemic. Women need to be heard to improve their own protective policies [51]. Hostels for female medical doctors on coronavirus duties and child-care stipends would help in reducing anxiety in women afraid of infecting their families. Additionally, media and community notables can support in raising awareness for symmetrical home management and alteration of regressive patriarchal values [53].

The Pakistan government’s response to physician’s requirement of personal protective equipment and safe work environment has been slow and disorganized [54]. Many hospitals in the country are catering to coronavirus patients and regular patients in the same premises, adding to the stress and predicament for physicians and other healthcare providers [55]. It has been shown that lack of personal protective equipment, sterilization, and active screening may also negatively influence perceptions of protection [56]. A study on preparedness conducted in India confirms that the majority of physicians believe their hospitals are not well prepared to confront the pandemics [57]. There is immediate need to improve perceptions and feelings of protection of physicians through state and social support.

The qualitative findings have been helpful in identifying the specific needs of physicians on coronavirus duty, and they may also have predictive influence on anxiety levels. The qualitative data also helps to explain much of the quantitative findings, specifically in relation to dissatisfaction with quality and completeness of personal protective equipment, problems with excessive workload and staff shortages, need for financial compensation and tax relief, and fear for families and children being exposed to the coronavirus. In addition, the needs indicated by the medical doctors highlight the increasing demands related to security and public support, and overall public health governance [58]. Some physicians indicated that their appointment in policy making committees to manage the pandemic was essential, as this would help plan protocols for sterilization of public spaces, lockdown logistics, and health literacy to disadvantaged populations [59]. Furthermore, there were other constructive demands for increased security, health worker team-building, and digital health service options to maintain physical distancing [60].

Finally, we must consider further studies for mental health, as frontline physicians exposed to coronavirus patients are also reporting depression, fear, sleeplessness, and stress [61]. The World Health Organization has encouraged that longitudinal and systematic assessment of the psychological needs of physicians, and other healthcare providers, working during the pandemic is needed [62]. Other research has suggested that coronavirus may be multiplying existing mental health problems among physicians [57, 63]. There is additional concern that due to social isolation and stigma associated with serving contagious and infectious populations, physicians may not be actively able to adopt health-seeking behaviors [64]. This would mean that physicians are entirely dependent on government and society to help them during this pandemic. We recommend online counseling and solidarity sessions and team-building for physicians and other health workers during these critical and uncertain times to improve emotional health.

### Limitations

Our study has certain limitations. We were unable to sample a larger number of respondents due to a low response rate. Low response rates are common for online-based surveys, but we may have missed physicians currently heavily involved in the corona pandemic due to their high workload. Nevertheless, the strength of this study is that it helps to identify factors that are contributing to anxiety in physicians working in coronavirus wards or centers in Pakistan. The findings of this study are important not just for Pakistan, but all developing nations with weak health systems combatting the coronavirus.

## Conclusions

The way in which the state and society support physicians, and other health workers, ultimately influences the national patient safety culture. The wellbeing and mental health of physicians, and other health workers, during global pandemics is also salient as it could impact on mortality and recovery rates. Immediate attention is needed to reduce anxiety, workload, and family strain in frontline practitioners treating coronavirus patients, and to improve their (perceptions of) protection. The wellbeing and safety of HCWs will determine the quality of preparedness for the next pandemic or the next wave of the corona pandemic. Collaborative efforts are needed by federal and provincial governments, the health sector, health regulatory bodies, media agents, and the public overall. Improving employment and financial benefits, staffing and resources, workplace safety and protective services, work family balance, security and governance participation, and team-building and emotional stability during these testing times will have long term benefits for physician service quality and patient safety standards.

## Supplementary Information

**Additional file 1: Supplementary file 1.** Questionnaire.

**Additional file 2: Supplementary file 2.** Factor analysis of study constructs.

## Data Availability

Data is available from corresponding author upon reasonable request.

## Abbreviations

CI: Confidence interval
COVID-19: Coronavirus Disease 19
M: Mean
PPE: Personal protective equipment
SD: Standard deviation
SPSS: Statistical Package for the Social Sciences
