# Supplementary material for: Anxiety amongst physicians during COVID-19: cross-sectional study in Pakistan
Source: BMC Public Health. 2021 Jan 11;21:118. doi: 10.1186/s12889-020-10134-4 (PMC7797886; doi:10.1186/s12889-020-10134-4)
Supplement: Supplementary file 1 — Additional file 1: Supplementary file 1. Questionnaire. [file 12889_2020_10134_MOESM1_ESM.pdf]

Challenges and Experiences of Pakistani Health Care Workers during COVID-19 Pandemic

COVER LETTER & ELECTRONIC INFORMED CONSENT

Dear Physicians,

First, Thank you for all that you are doing for us! We pray for your safety and wellbeing.

Second, we request you to please take a few minutes time to help us complete this survey. It is targeted to help improve public health policy and social policy to protect you and your families in the future.

Your complete and honest answers will help us to recommend and mobilize suitable policy at federal and provincial levels.

Thank you,

Researchers from Forman Christian College University, International Islamic International University Islamabad, and University of Lahore.

For questions:
Dr. Sara Rizvi Jafree
Assistant Professor, Department of Sociology
Forman Christian College University
Email: sarajafree@fccollege.edu.pk Cell: 0300 400 5740

Electronic Tab for Informed Consent _____________
Name of coronavirus ward/ isolation center
*(This information is confidential and will not be part of the publication)*____________
City
*(This information is confidential and will not be part of the publication)*______________

QUESTIONNAIRE

Age :	up to 30 years	31-35	36-40	41-45
	46-50	51-55	56-60	above than 60 years
Gender:	Male	Female		

Job Specification:

Doctor Nurses Ward Boys Radiological/ Lab Technician Pharmacist

Please list your employment designation:____________________

(E.g.- Junior doctor, Assistant Professor, Nurse superintendent, ward nurse, trainee…)

Job Experience (In Years):

Current Working Place: Outdoor (OPD)

Emergency

Ward General

Ward

Quarantine Centers made for COVID-19 patients
Isolations Centers made for COVID- 19

patients Intensive Care Unit (ICU) for

COVID- 19 patients Other, please specify: ________________________________

Did you receive personal protective equipment (PPE) during COVID-19 outbreak?

Yes provided by the government

officials Yes arranged by the Hospital

Administration

Yes arranged by doctors’ representative bodies

Yes donated by NGOs

Not received yet.

Are you regularly receiving N-95 masks when you are on duty?

Always Often Sometimes Rarely Never

Are you regularly receiving surgical masks when you are on duty?

Always Often Sometimes Rarely Never

		Never	Rarely	Sometimes	Often	Always
Please answer these questions in relation to novel coronavirus						
1	I feel anxious about being infected by the virus					
2	I feel anxious about compensation, in the case of being infected					
3	I feel hesitation in working					
4	I feel I have no choice but to work due to obligation					
5	I feel anxious about being infected during commuting/travel to work					
6	I feel I have incomplete knowledge about prevention and protection from this virus					

7	I feel I have lack of knowledge about coronavirus infection.					
Please answer these questions in relation to your work energy and motivation to work						
	I feel elevated in mood					
	I have insomnia					
	I am exhausted physically					
	I am exhausted mentally					
	I feel burdened by the increase in quantity of work					
	I feel burdened by the changed nature of work					
	I feel it's my professional responsibility to save lives					
	I want to get my country out of this national crisis.					
	I feel it's time to sacrifice for humanity					
	Through my work I feel satisfied in front of my God					
Please answer these questions in relation to your perceived protection and support						
	I feel I am protected by the federal government					
	I feel I am protected by my hospital administration					
	I feel I am protected by security forces					
	My friends are appreciating my work commitment					
	My colleagues are supporting and encouraging me to continue working					
	My family members are happy and praying for me while I am working					
	People pay tribute (saluting, clapping, and waving of hands) to me when I go outside from the hospital					
	People pay tribute (praise, commend) us (healthcare workers on duty) on social media.					
Please answer these questions in relation to family strain						
	I am worried about returning home and exposing my children to virus.					

	I am worried I carry the virus without symptoms, and place my parents at risk.					
	I feel anxious about my family whenever see any patient in critical situation.					
Please answer these questions in relation to religiosity						
	I pray to God whenever go to hospital.					
	I feel that God will help us to get out of these difficult times.					
	I keep praying to God while treating/attending any suspected or confirmed patient of coronavirus.					

What are the top three coping strategies that you have adopted while working during this pandemic?

What are the top five things that you require from the government/ your employer in this time?

How do you think healthcare workers can be better trained and supported in the future for such crises?

