# Supplementary material for: Anxiety amongst physicians during COVID-19: cross-sectional study in Pakistan
Source: BMC Public Health. 2021 Jan 11;21:118. doi: 10.1186/s12889-020-10134-4 (PMC7797886; doi:10.1186/s12889-020-10134-4)
Supplement: Supplementary file 2 — Additional file 2: Supplementary file 2. Factor analysis of study constructs. [file 12889_2020_10134_MOESM2_ESM.pdf]

<u>Factor analysis</u>

**Stressors**	Component			
	F1	F2	F3	F4
Factor 1 : Exhaustion (Cronbach a = 0.878)				
I am exhausted physically.	**.862**	.121	.065	.223
I feel burdened by the changed nature of work.	**.838**	.250	.018	.108
I am exhausted mentally.	**.774**	.082	.108	.248
I feel burdened by the increase in quantity of work.	**.774**	.040	.228	.117
I have insomnia.	**.659**	.230	.269	-.223
Factor 2: Family Strain (Cronbach a = 0.791)				
I am worried I carry the virus without symptoms, and place my family (children and parents) at risk.	.161	**.849**	.062	.202
I am worried about returning home and exposing my children to virus.	.133	**.836**	-.042	.263
I can't stop worrying for my family whenever I see/treat a patient in critical situation.	.139	**.730**	.016	-.069
Factor 3: Workload (Cronbach a = 0.725)				
I feel I have lack of knowledge about coronavirus infection.	.015	-.085	**.865**	.112
I feel I have incomplete knowledge about prevention from this virus.	.197	-.116	**.863**	.057
I feel I have no choice but to work due to obligation.	.115	.276	**.524**	.336
I feel hesitation in working.	.323	.275	**.515**	.046
Factor 4: Anxiety (Cronbach a = 0.644)				
I feel anxious about being infected during commuting/travel to work.	.049	.135	.014	**.785**
I feel anxious about compensation, in the case of being infected.	.254	.053	.331	**.619**
I feel anxious about being infected by the virus.	.374	.384	.272	**.493**
Eigenvalue	5.31	2.03	1.59	1.02
Variance Explained (%)				66.49

**Motivators**	Component			
	1	2	3	4
Factor 1: Work Commitment (Cronbach a = 0.792)				
I feel it's time to sacrifice for humanity.	**.786**	.038	.130	.003
I feel it's my professional responsibility to save lives.	**.779**	.154	-.088	.083
My family members are happy and praying for me while I am working.	**.742**	.098	.102	.134
I want to get my country out of this national crisis	**.724**	.112	-.157	.040
My friends are appreciating my work commitment.	**.670**	.106	.082	.138
Factor 2: Spirituality (Cronbach a = 0.795)				
I feel that God will help us to get out of these difficult times.	.071	**.865**	-.039	-.092
I pray to God whenever I go to hospital/ workplace	.180	**.796**	-.063	.216
I keep praying to God while treating/attending any suspected or confirmed patient of coronavirus.	.087	**.795**	-.035	.139
Through my work I feel satisfied in front of my God.	.498	**.578**	.267	-.019
Factor 3: Feeling of being protected (Cronbach a = 0.796)				
I feel I am protected by my hospital administration.	.023	-.051	**.873**	.095
I feel I am protected by the federal government.	-.009	-.003	**.828**	.079
I feel I am protected by security forces.	.079	.008	**.734**	.357
Factor 4: Sense of acknowledgment (Cronbach a = 0.709)				
People pay tribute (saluting, clapping, and waving of hands) to me when I go outside from the hospital/ workplace	.193	.125	.160	**.843**
People pay tribute (praise, commend) us (healthcare workers on duty) on social media.	.081	.072	.237	**.818**
Eigenvalue	4.23	2.54	1.77	1.09
Variance Explained (%)				64.34
